# Supplementary material for: Computational method for aromatase-related proteins using machine learning approach
Source: PLoS One. 2023 Mar 29;18(3):e0283567. doi: 10.1371/journal.pone.0283567 (PMC10057777; doi:10.1371/journal.pone.0283567)
Supplement: Attachment — Submitted filename: Response to reviewers.doc [file pone.0283567.s002.doc]

Dear Editor,

Thank you for providing us an opportunity to submit a revision of our manuscript. Below is a point-by-point response to all the reviewers. All the changes in the revised manuscript are highlighted in yellow.

**Reviewer #1:**

The manuscript sounds interesting, and the authors have developed method to predict the aromatase-related proteins. The manuscript is well structured and the authors have elucidated their method distinctly. However, there are some corrections could be made to make it more precise and clear;

1- The abstract should be more organized and includes briefly the method, result, and conclusion.

Yes, we have modified the abstract as per your guidance (highlighted in yellow).

2- The last paragraph of the introduction should be moved to the method section.

Yes, some part of the paragraph is now moved to the Method section on page 4 as para 1 (highlighted in yellow).

3- There are some out dated references, I recommend the authors to use most recent ones.

Yes, now we have added the recent references (highlighted in yellow).

**Reviewer #2:**

Dear authors,

Your manuscript is highly interesting, and it caught my interest as I wrote almost same journal article after a regress work on SARS CoV-2. However, your manuscript is having a huge flaw, the Abstract, methods, and conclusion are totally not in the article fashion, improvise it properly as you have done wonderful work.

Yes, we have modified the abstract, methods and conclusion as per your guidance (highlighted in yellow).

**Reviewer #3:**

Dear Authors,

Thank you for submitting your work to Plos One journal. Some improvements need to be considered to make your work looks better.

Please consider the following comments and suggestions:

1. **Abstract**:

a. I suggest moving the link to the related section (dataset).

Yes, we have moved the link to webserver in dataset section in the methods on page 5 (highlighted in yellow).

b. Please recheck the link and make sure it is correct and working.

Due to some security reason the provided link was not working, now we have secured our site under (https). Now it can be accessed.

2. **Introduction**:

Please follow the traditional way of writing this part. For instance, at the end of this part you should mention the structure of your article.

Yes, we have modified the end of Introduction as per your guidance (highlighted in yellow).

3.**Methods**:
- Datasets for SVM: you mentioned removing sequences labeled "fragments,", "isoforms",.. etc. Please elaborate.

Yes, it is now explained in the Dataset for SVM section on page 5 (highlighted in yellow).

- Generation of survival curves: please explain more about Kaplan-Meier tool.

Yes, the explanation is now added to the section on page 4 (highlighted in yellow).

4. **Results**:

- In table 1: The p-value numbers varies a lot! for example the p-value for Esophageal Adenocarcinoma on RFS was 0.93 while o-value for Head-neck squamous cell carcinoma was 0.048. Please explain the reason.

The p-value is calculated by the KM plotter website for each cancer type and hence it is significant for some types of cancers and not significant for others.

5. **Conclusion**:

Based on your findings, do you suggest any future work?

Yes, we are interested to work on molecular docking of aromatase inhibitors.

- Please check the equations and formulas because it appears that there's something wrong with the fraction bars!

Yes, we have corrected the equations and formulas.

**Reviewer #4:** The present study aims to develop a method to recognize aromatase protein using different amino acid composition parameters and evolutionary profile using SVM technique. Also, the authors state that they have developed a webserver for the same.
However, from the introduction given in the manuscript it is difficult to understand that why authors need to predict Aromatase protein.

There are many unknown functional proteins, which are available and need to be identified. Thus, there is a need of computational methods to identify these proteins and their functions. So, we have developed a method to identify these aromatase-related proteins.

They state that Aromatase is a target for designing inhibitors in humans for treating Cancer and other diseases. But, then the sequence of this protein is already known. So what is the purpose of this study. Also, if I understand Aromatase is one single protein and if we start developing methods for single proteins then we can develop many thousand methods. But what is the utility?

It is not like that; we have used aromatase as one of the major enzymes in the cancer-related study. So, we developed a computational method which will be useful for the cancer researchers. Actually, it is one of the major tasks in bioinformatics to predict the protein functions which can assist with a variety of biological issues, such as understanding disease mechanisms or identifying novel drug targets.

**Minor comments**

1. In “Method” section, number of sequences initially taken from Uniprot/Swiss-prot as well as number of sequences removed to get a final dataset of 191 sequences is not mentioned.

Yes, when we used the keyword “aromatase”, we found 9836 protein sequences which included 257 reviewed sequences. We only used reviewed sequences and the final dataset contained 191 sequences out of 257. This is added on page 5 under “Datasets for SVM” (highlighted in yellow).

2. Have the authors compared the similarity of negative set sequences with positive dataset?

We have not done the similarity of negative set sequences with positive dataset. The negative sequences are totally different from positive set sequences which were selected using keyword in the uniprot databases.

3. Amino acid profiles of aromatase and non-aromatase was found to be similar by authors (Fig 2B). Then how it has contributed in model development

Sorry, some of the residues patterns are similar, but not all. These differences can identify the aromatase from negative sequence by the developed models.

4. The web link given for the server could not accessed.

Due to some security reason the provided link was not working, now we have secured our site under (https). Now it can be accessed.
