# Supplementary material for: Computational method for aromatase-related proteins using machine learning approach
Source: PLoS One. 2023 Mar 29;18(3):e0283567. doi: 10.1371/journal.pone.0283567 (PMC10057777; doi:10.1371/journal.pone.0283567)
Supplement: Attachment — Submitted filename: Response to reviewers-25.2.23.doc [file pone.0283567.s003.doc]

Dear Editor,

Thank you for providing us an opportunity to submit a revision of our manuscript. Below is a point-by-point response to the reviewer. All the changes in the revised manuscript are highlighted in yellow.

**Reviewer #2:**

1. Make a graphical abstract (Flow chart) for the complete study how you conceptualized to what you obtained followed by each method in depth. [https://www.ncbi.nlm.nih.gov/core/lw/2.0/html/tileshop_pmc/tileshop_pmc_inline.html?title=Click%20on%20image%20to%20zoom&p=PMC3&id=6386390_pcbi.1006730.g001.jpg](https://www.ncbi.nlm.nih.gov/core/lw/2.0/html/tileshop_pmc/tileshop_pmc_inline.html?title=Click on image to zoom&p=PMC3&id=6386390_pcbi.1006730.g001.jpg))

Yes, we have prepared a graphical abstract as per your guidance, which is now Figure No. 6.

2. Kindly make the source code available through GitHub with proper documentations.

It is really good to keep our web-tool in the GitHub and we will try it in our future studies. At present, our institute (CSIR-IMTECH) has provided all the facility to host our web-tool and it is also being a part of an open source portal.

3. The server link is not working, make sure your institute keep it live.

Yes, our server is on live at <https://bioinfo.imtech.res.in/servers/muthu/aromatase/submit.html>

4. You have used different SVM approach-based models with amino acid, dipeptide composition, hybrid and evolutionary profiles to predict. This is good; however, the tool must work with it in background and in the front the user only supposed to see the results and if user wants there supposed to be a button to view the documentation how and why the SVM has classified them to be aromatase.

Yes we agree, but it is not possible to show all the background running programs. Actually, it has a set of programs wrote in perl script. All the programs were kept in cgi-bin folder, which run only in the background and it is not for public viewing. The output results will be displayed in a new web page.

From documentation point of view, it is a good idea that the user can view the complete results. We will try it in our future studies.

5. In methods - Provide the dataset in supplementary files and keep the search date to not make it irrelevant for future researchers.

Yes, we have provided all the dataset as a supplementary file-1. The retrieval date is now mentioned in the dataset section (highlighted in yellow).

6. The Introduction and discussion is not sufficiently written, it needed to be validated with any other expert, authors can read some more articles to understand how to write introduction as well as discussion.

Yes, we have modified the Introduction and discussion as per your guidance (highlighted in yellow).
